# Research on a 3D Encapsulation Technique for Capacitive MEMS Sensors Based on Through Silicon Via †

**DOI:** 10.3390/s19010093

**Published:** 2018-12-28

**Authors:** Meng Zhang, Jian Yang, Yurong He, Fan Yang, Fuhua Yang, Guowei Han, Chaowei Si, Jin Ning

**Affiliations:** 1 Institute of Semiconductors, Chinese Academy of Sciences, Beijing 100083, China; zhangmeng@semi.ac.cn (M.Z.); yangjian@semi.ac.cn (J.Y.); yangfan3104@semi.ac.cn (F.Y.); fhyang@semi.ac.cn (F.Y.); 2 College of Materials Science and Opto-Electronic Technology, University of Chinese Academy of Sciences, Beijing 100049, China; hyr617@semi.ac.cn; 3 School of Microelectronics, University of Chinese Academy of Sciences, Beijing 100049, China; 4 School of Electronic, Electrical and Communication Engineering, University of Chinese Academy of Sciences, Beijing 100049, China; 5 State Key Laboratory of Transducer Technology, Chinese Academy of Sciences, Beijing 100083, China

**Keywords:** 3D encapsulation, capacitive, MEMS, vertical interconnect, glass reflow

## Abstract

A novel three-dimensional (3D) hermetic packaging technique suitable for capacitive microelectromechanical systems (MEMS) sensors is studied. The composite substrate with through silicon via (TSV) is used as the encapsulation cap fabricated by a glass-in-silicon (GIS) reflow process. In particular, the low-resistivity silicon pillars embedded in the glass cap are designed to serve as the electrical feedthrough and the fixed capacitance plate at the same time to simplify the fabrication process and improve the reliability. The fabrication process and the properties of the encapsulation cap were studied systematically. The resistance of the silicon vertical feedthrough was measured to be as low as 263.5 mΩ, indicating a good electrical interconnection property. Furthermore, the surface root-mean-square (RMS) roughnesses of glass and silicon were measured to be 1.12 nm and 0.814 nm, respectively, which were small enough for the final wafer bonding process. Anodic bonding between the encapsulation cap and the silicon wafer with sensing structures was conducted in a vacuum to complete the hermetic encapsulation. The proposed packaging scheme was successfully applied to a capacitive gyroscope. The quality factor of the packaged gyroscope achieved above 220,000, which was at least one order of magnitude larger than that of the unpackaged. The validity of the proposed packaging scheme could be verified. Furthermore, the packaging failure was less than 1%, which demonstrated the feasibility and reliability of the technique for high-performance MEMS vacuum packaging.

## 1. Introduction

Encapsulation is crucial for microelectromechanical systems (MEMS) sensors, since it can shield the sensitive and fragile structures from the influence of the external environment to obtain a high performance. On account of the feature of customization, that the packaging requirements for various MEMS sensors are different, the packaging cost accounts for over 50% of the total fabrication cost [1]. Therefore, choosing a suitable packaging solution is significant in order to obtain better performance and lower costs. The 3D wafer-level packaging technology, combined with a wafer bonding and vertical interconnection technique, has great potential in realizing a smaller size, lower power consumption, and lower fabrication cost [2,3,4,5,6]. Due to the fact that Pyrex glass has a similar coefficient of thermal expansion (CTE) to silicon, the anodic bonding between silicon and glass is usually applied to the hermetic packaging for MEMS devices to obtain high packaging reliability. Considering the cost, reliability, and efficiency, anodic bonding is optimal for MEMS sensors compared with a commercially available eutectic bonding technique. Thus, it is significant to design the packaging process of MEMS sensors based on anodic bonding for lower cost.

In addition to packaging hermeticity, the electrical interconnection is also indispensable to transmit an electrical signal which responds to external physical quantities. Vertical interconnection techniques can reduce delay and power consumption due to shorter interconnection lengths [7] and improve the integrated level, space efficiency, and design simplicity [8]. Compared with lateral feedthrough with the problems of step coverage, it is easier to realize good hermetic encapsulation with the vertical feedthrough [9]. The vertical interconnection structures fabricated by a Pyrex glass reflow process, where the low-resistivity silicon pillars surrounded by the insulated glass act as the medium to transmit an electrical signal, has the advantages of good isolation, negligible parasitics, and low crosstalk [10], in addition to good compatibility with anodic bonding. The technology has been widely applied to MEMS pressure sensors [11,12], radio frequency (RF) resonators [12,13,14], and gyroscopes [15]. 

However, in plane-parallel capacitive MEMS sensors, the patterned metal layer or polysilicon in an etched glass cavity are generally used as the fixed capacitance plates [11,12,16]. The issues regarding the adhesion between the metal and etched glass surface, and the thermal stress caused by CTE mismatch are inevitable. Furthermore, the size of the fixed capacitance plate is often larger than wanted due to the design margin for lateral leads, which will degenerate the performance and reliability of the MEMS sensors.

**Figure 1:**
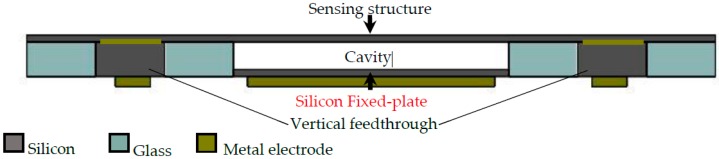
Cross-sectional schematic for the packaged capacitive microelectromechanical systems (MEMS) sensor.

In this paper, an improved 3D hermetic packaging scheme based on anodic bonding for capacitive MEMS sensors is studied, as shown in Figure 1. We use the embedded silicon pillars, instead of metal or polysilicon, as the fixed capacitance plate to simplify the process and improve the reliability. It is the third function of the encapsulation cap, except for realizing hermetic sealing and vertical electrical interconnection. The silicon wafer with sensing structures is anodically bonded to the encapsulation cap at the wafer level to form the sensing capacitor. The sensing structures deform with the change of external physical quantities, resulting in the variation in the capacitance value. The packaging scheme can be designed flexibly for various capacitive MEMS sensors, such as pressure sensors, accelerators, gyroscopes, ultrasonic transducers, and so on. The fabrication process flow of the encapsulation cap based on a GIS process is introduced in detail. The surface morphology and the electrical property of silicon vertical feedthrough were also characterized. Finally, a capacitive gyroscope was successfully fabricated with the proposed packaging scheme. The quality factors of the unpackaged gyroscope and packaged gyroscope were both measured to evaluate the validity and reliability of the proposed encapsulation scheme.

## 2. Fabrication of Encapsulation Cap

**Figure 2:**
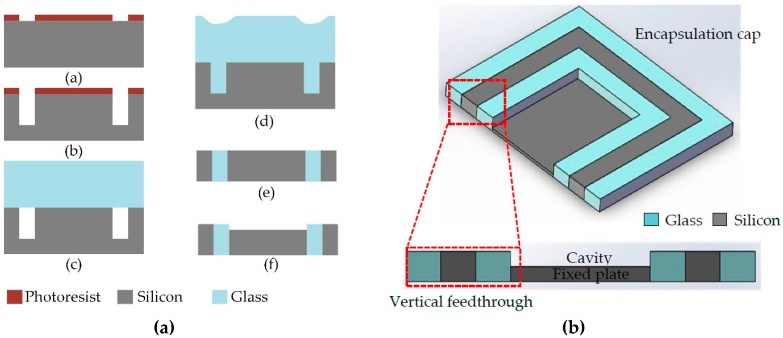
(**a**) Process flow for the encapsulation cap; (**b**) schematic and the sectional view of the encapsulation cap.

The fabrication process flow of the encapsulation cap with through silicon vias (TSVs) is shown in Figure 2a, which is presented in detail as follows.

(a) A 4-inch silicon wafer with a low resistivity (0.0009 Ω·cm) was used to realize a low loss electrical interconnection. The substrate was patterned by photolithography and an 8-μm thick photoresist acted as the mask for deep silicon etching.
(b) The mature deep reactive ion etch (DRIE) process was utilized to etch silicon of 350 μm to form silicon molds for the glass reflow process. The photoresist was removed and the chemical cleaning of the wafer was carefully conducted for the following anodic bonding.
(c) The silicon substrate was bonded to the Pyrex glass wafer in a vacuum environment. The silicon substrate and the glass wafer had the same thickness and a similar coefficient of thermal expansion (CTE) to minimize the warp and distortion of the bonded wafer. It should be noted that it is necessary to hold the vacuum conditions of 10^−2^ mTorr (1 Torr ≈ 133 Pa) for enough time to achieve vacuum outgassing, which can prevent generating voids during the glass reflow process.
(d) The bonded wafer was then placed in the furnace at 1050 °C for 2 h to ensure that the silicon molds were fully filled with the fused glass. When the temperature was elevated above 750 °C, the fused glass started to be pushed into the silicon molds under the large pressure difference between inside and outside the hermetic cavity. Subsequently, the glass was cooled by a cooling process.
(e) The reflowed wafer was lapped and thinned on double sides until the formation of the silicon vertical feedthrough. A chemical mechanical polishing (CMP) process was exploited to provide good surface quality for later wafer bonding.
(f) To form the encapsulation cavity, the silicon was etched by an Inductively Coupled Plasma (ICP) process to a certain depth according to design requirements. The cavity acted as the gap of the sensing capacitor when the following anodic bonding was accomplished.

The schematic of the encapsulation cap is shown in Figure 2b. The silicon feedthrough structure runs through the wafer from top to bottom just as its name implies. The silicon vertical feedthrough minimizes the routing lengths for electrical signals between the substrates, which can realize a smaller footprint and lower power consumption [12]. In particular, some of the silicon pillars surrounded by glass serve as the fixed capacitance plates and are etched to form the packaging cavity after anodic bonding. The lateral electrical feedthrough with a certain thickness may become the leakage path through the bonding interface. It is easier to realize hermetic encapsulation with the vertical feedthrough. This is the reason why the vertical electrical interconnects are adopted in our packaging scheme.

## 3. Characterization and Measurement

**Figure 3:**
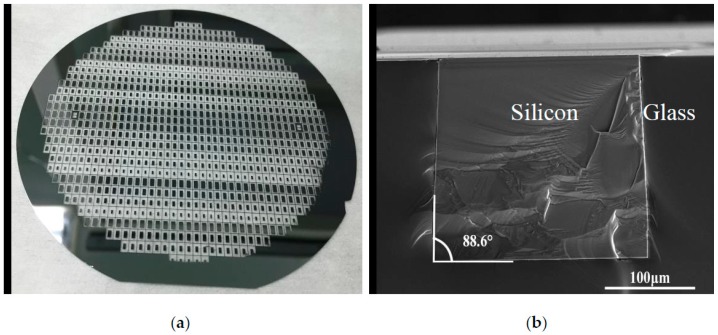
(**a**) Photograph of the capping wafer; (**b**) Cross-sectional SEM view of the silicon vertical feedthrough structure.

The accomplished capping wafer is shown in Figure 3a. It can be seen that the silicon feedthrough structures, which have been fabricated in different sizes and shapes, are surrounded by glass. Figure 3b shows the scanning electron micrograph (SEM) for the cross-section of silicon feedthrough embraced by reflowed glass. No voids are found in the reflowed glass and even at the silicon-glass interface, which means that the glass reflow process parameters are reasonable and the reflowed glass can provide good insulation. In addition, the silicon etching sidewall is nearly perpendicular to the substrate with an angle of 88.6°. The good etching profile is attributed to mature deep silicon etching technology. In comparison with the tapered via, the thermal stress concentration as a result of nonuniformity can be alleviated greatly in the vertical via [17], obtaining good thermal stability and high reliability.

**Figure 4:**
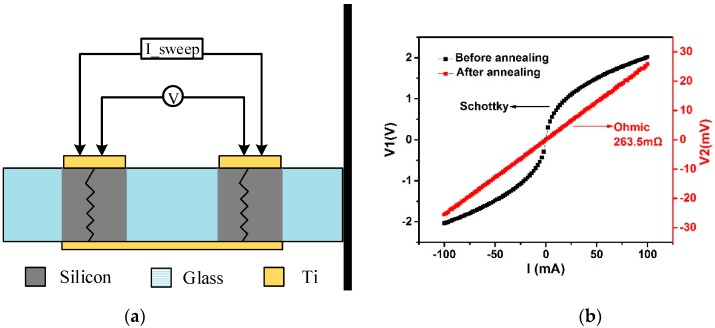
(**a**) Testing schematic using a four-probe method; (**b**) The voltage–current testing results using B1500A. The black curve was acquired before annealing and the red curve was acquired after annealing.

To characterize the electrical property of the silicon vertical feedthrough directly and effectively, the resistances were measured by a four-probe method which could eliminate the parasitic effect of the testing system. The sample was placed on the probe station and the sweep signals from B1500A were imposed on the metal electrodes by probes. The testing schematic of the experimental sample is shown in Figure 4a. Two testing loops were obtained, in which one loop offered the sweep current and the other loop worked equivalently to a voltmeter. Thus, the relationship between the voltage (V) and the sweep current (I) across the testing samples could be obtained. 

It should be noted that the Schottky junction exists between metal and silicon, which is unfavorable for resistance measurement. The thermal annealing is generally performed for the purpose of obtaining the ohmic contact. However, good ohmic contact can be also achieved during anodic bonding because the wafer can be simultaneously thermally annealed by the high bonding temperature of 400 °C. It means that good ohmic contact can be realized without extra process and that the packaging design is totally compatible with the fabrication flow of MEMS sensors. The measurement results obtained before and after annealing are shown in Figure 4b. The results indicate clearly that good ohmic contact was achieved, with the resistance calculated as 263.5 mΩ from the slope of the voltage–current curve, which was almost as low as the resistance of copper feedthrough [18]. Good electrical interconnection was realized and the feasibility to use an embedded silicon feedthrough as the fixed capacitance plate was also validated at the same time.

The encapsulation cap and silicon wafer need to be bonded together to realize 3D wafer-level encapsulation. Prior to the wafer bonding, the surface roughness after polishing was characterized to evaluate the feasibility of wafer bonding. The roughness will affect the packaging hermeticity. During anodic bonding, the plastic deformation occurs to the glass at the bonding temperature of 400 °C to ensure the close contact between glass and silicon at the interface. Since the plastic deformation of glass is limited, the hermeticity will become worse with the increase of surface roughness in a certain range. However, anodic bonding is more tolerant of roughness on the bonding surface compared to a fusion bonding technique [19].

**Figure 5:**
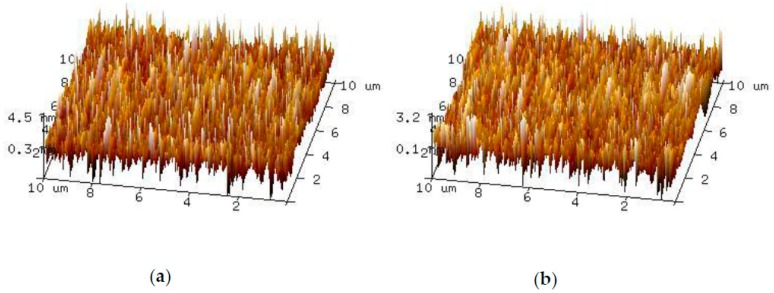
The roughness measurement results obtained using an atomic force microscope (AFM) after polishing (**a**) the measurement result for glass surface; (**b**) the measurement result for silicon surface.

The surface roughness was measured under a scanning range of 10 μm × 10 μm by an atomic force microscope (AFM) in tapping mode. The scanning results of glass and silicon are respectively exhibited in Figure 5a,b. The RMS roughnesses of glass and silicon were 1.120 nm and 0.814 nm, respectively, values which were much smaller than the surface roughness requirement of 5 nm for anodic bonding [20] and the surface was smooth enough for anodic bonding.

## 4. Application and Discussion

**Figure 6:**
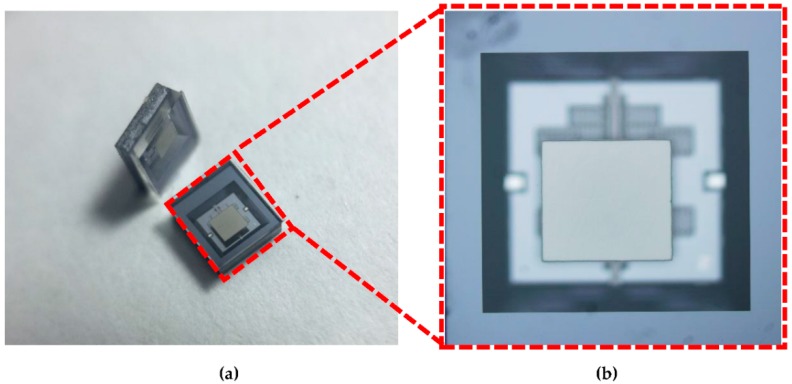
(**a**) The vacuum packaged gyroscopes; (**b**) close-up view of a gyroscope

Finally, the encapsulation scheme was applied to a capacitive gyroscope to evaluate the validity. The silicon wafers prefabricated with sensing structures were anodically bonded in a vacuum at the wafer level with two encapsulation caps to form a sandwich structure. Figure 6a shows two packaged gyroscopes with the size of 3 mm × 3 mm and the enlarged view of the device is also given in Figure 6b.

The sensing capacitor consisted of the movable sensing structure and two silicon fixed plates embedded in the caps while the encapsulation cavities served as the capacitance gaps. The input angle velocity caused the displacement of the movable sensing structure owing to the Coriolis Effect and was exhibited as the variation in the capacitance.

Vacuum packaging is done for the air dumping control which is critical for a high-performance gyroscope. The gyroscope could be equivalent to a second-order underdamped system, whose motion equation is given as follows [1].

(1) $$m\frac{d^{2}A\left(t\right)}{dt^{2}}+c\frac{dA\left(t\right)}{dt}+kA\left(t\right)=0$$

where A(t) is the instantaneous displacement of the sensing structure from the equilibrium position, m is the mass, k is the spring stiffness, and c. is the damping factor which describes the influence of damping to the vibration of the sensing structure.

A(t) can be derived as Equation (2) [1] by solving the above second-order differential equation, which describes the vibration of the sensitive structure.

(2) $$A\left(t\right)=A_{0}e^{-\lambda t}\sin\left(\sqrt{w_{r}{}^{2}-\lambda^{2}}t+\varphi_{0}\right)\left(w_{r}{}^{2}\gg \lambda^{2}\right)$$

(3) $$w_{r}=\sqrt{\frac{k}{m}}$$

where $A_{0}$ is the initial amplitude, $\varphi_{0}$ is the initial phase, λ is the ratio of c/2m, whose inverse $\frac{1}{\lambda }$ stands for the relaxation time, and $w_{r}$ is the resonant angle frequency of the gyroscope and can be calculated according to Equation (3) [1]. From Equation (2), it can be seen that the vibrating amplitude, $A_{0}e^{-\lambda t}$, decreases exponentially along with the time caused by the damping effect. If the damping factor c is zero, the gyroscope can make periodic back-and-forth motions at a constant amplitude, which means the energy stored in the gyroscope remain unchanged. Thus, the damping effect, especially air damping, is the main factor that causes energy dissipation.

To evaluate the encapsulation performance, mainly the damping effect, the quality factor (Q) of the gyroscope was characterized, which is defined as the ratio of the energy stored in a device to the energy dissipated per cycle of resonance [21], as shown in Equation (4). A high quality factor indicates low rate of energy dissipation, namely, low damping.

(4) $$Q=2\pi \frac{U_{Store}}{U_{Disspate\;per\;cycle}}=2\pi \frac{U_{t=t_{0}}}{U_{t=t_{0}}-U_{t=t_{0}+T}}$$

where U stands for the energy, t is the time, and T is the vibration period. The energy U can be calculated by the Formula (5) [1]. A is the vibration amplitude of the sensing structure, which is the maximum displacement from the equilibrium position.

(5) $$U=\frac{1}{2}kA^{2}$$

By substituting Equations (2) and (5) into the Equation (4), Q can be derived as shown in Equation (6) [21], where $f_{r}$ is the natural resonant frequency of the gyroscope. Thus, the relaxation time $\frac{1}{\lambda }$ and the resonant frequency $f_{r}$ are necessary to determine the quality factor of the gyroscope.

(6) $$Q=\frac{\pi }{\lambda T}=\frac{\pi }{\lambda }*\frac{\sqrt{w_{r}{}^{2}-\lambda^{2}}}{2\pi }\approx \pi \frac{1}{\lambda }\frac{w_{r}}{2\pi }=\pi \frac{1}{\lambda }f_{r}$$

**Figure 7:**
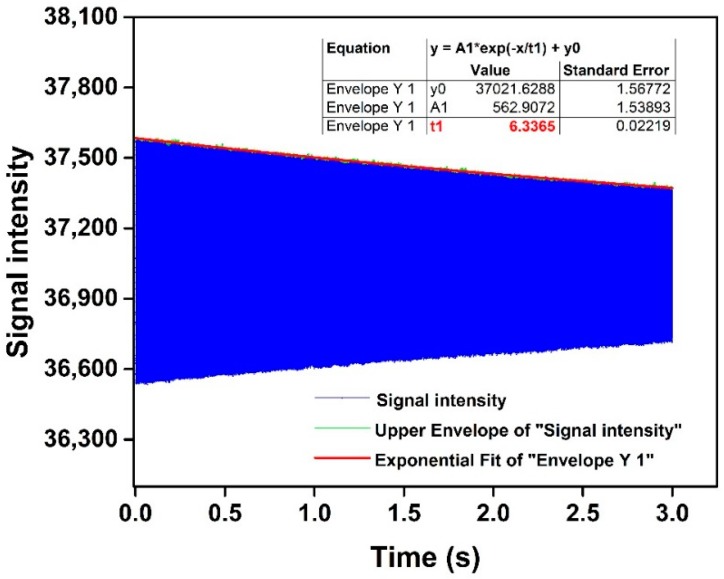
The time–domain dynamic response measured at the drive mode of the gyroscope.

Here, the quality factor was determined by studying the free vibrations of the gyroscope in the time domain. The sample frequency was 31.25 kHz. The signal intensity during free vibration as a function of time was measured, see the blue curve in Figure 7. The signal intensity reflected the vibration of the sensing structures. The green curve shows the vibration amplitude as a function of time obtained by the upper envelope fitting. It could be seen that the signal intensity gradually decreased over time, in accordance with the theory. The relaxation time $\frac{1}{\lambda }$ of 6.3365, can be acquired by an exponential fit of the vibration amplitude.

**Figure 8:**
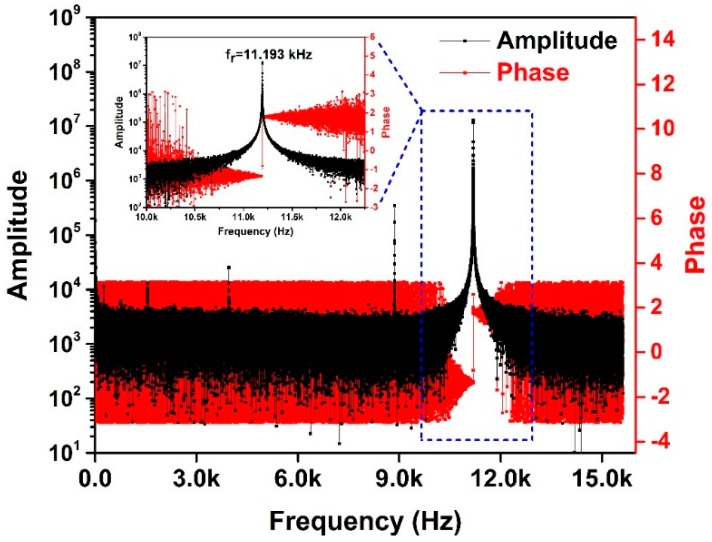
The frequency–domain characteristics of the gyroscope obtained by Fast Fourier Transform (FFT), in which the black is the amplitude–frequency curve and the red is phase–frequency curve.

To obtain the resonant frequency $f_{r}$ of the gyroscope, the Fast Fourier Transform (FFT) was performed for the dynamic response in the time domain with the MATLAB software. The frequency–domain characteristics can be calculated, as shown in Figure 8. The resonant frequency point can be easily determined to be 11.193 kHz by observing the amplitude–frequency curve and phase–frequency curve.

The quality factor of the gyroscope at the driving frequency of 11.193 kHz can be calculated as 222,815 according to the Formula (6). The high quality factor means low air damping, which indicates a high vacuum degree of the packaging cavity.

To further evaluate the pressure level in the encapsulation cavity and the validity of the packaging method, the resonant characteristics of the gyroscopes before packaging were also measured by a Laser Doppler Dynamic Signal Analyzer. The gyroscope to be tested was fixed in the chamber of the vibration station. The motion of the sensitive structures under the periodic chirp excitation could be recorded by the laser.

(7) $$Q=\frac{f_{r}}{\Delta f_{3dB}}$$

**Figure 9:**
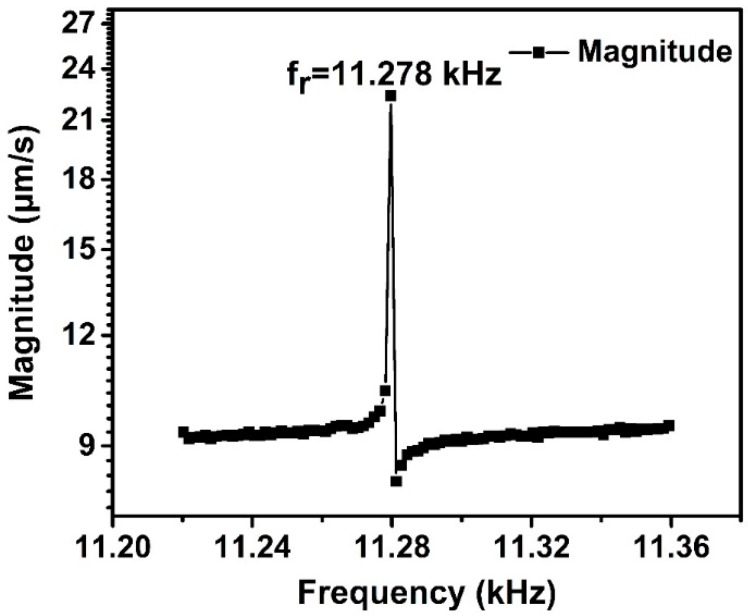
The resonant characteristics at the drive mode of the unpackaged gyroscopes at a pressure of 3 Pa measured by a Laser Doppler Dynamic Signal Analyzer.

The resonant characteristics of the gyroscope were obtained, as shown in Figure 9, when the air pressure in the chamber was stabilized at 3 Pa. The quality factor for the unpackaged gyroscope was calculated as 13,313 by the Formula (7) [21], which is much smaller than the quality factor of 220,000 for the packaged gyroscope. $\Delta f_{3dB}$ is the 3-dB bandwidth, namely, the corresponding bandwidth when the gain is 3 dB less than the mid-band gain. 

Compared with the quality factor for the unpackaged gyroscopes, the quality factor increased by one order of magnitude at least by adopting the proposed packaging scheme. The pressure in the packaged cavity could be evaluated below 1 Pa (10^−2^ mbar) according to the theoretical model for quality factor [22], which lies in the required vacuum range from 10^−1^ to 10^−4^ mbar for MEMS gyroscopes [23]. The validity of the proposed packaging scheme can be verified. Meanwhile, the feasibility and reliability of the packaging scheme were also demonstrated by the fact that the packaging failure is less than 1% and the air pressure in the cavity remained nearly unchanged even after 700 days.

## 5. Conclusions

A 3D hermetic packaging technique suitable for capacitive MEMS sensors is studied. The encapsulation cap with a silicon vertical feedthrough and good surface quality was successfully fabricated and applied to the encapsulation of a capacitive gyroscope. Good electrical interconnection was realized by using the silicon vertical feedthrough. The feasibility to use the silicon pillars embedded in the cap, instead of metal or polysilicon, as the fixed capacitance plate was also validated, which could simplify the process and improve the reliability. The quality factor was enhanced by one order of magnitude by adopting the proposed encapsulation scheme, which proved the validity of the scheme. The feasibility and reliability of the 3D vacuum packaging technique were demonstrated by the successful application to MEMS gyroscopes. The packaging scheme can be designed flexibly to control the vacuum degree for various capacitive MEMS sensors to realize high performance.
